# Realness of face images can be decoded from non-linear modulation of EEG responses

**DOI:** 10.1038/s41598-024-56130-1

**Published:** 2024-03-07

**Authors:** Yonghao Chen, Tilman Stephani, Milena Teresa Bagdasarian, Anna Hilsmann, Peter Eisert, Arno Villringer, Sebastian Bosse, Michael Gaebler, Vadim V. Nikulin

**Affiliations:** 1 Department of Neurology, Max Planck Institute for Human Cognitive and Brain Sciences, Leipzig, Germany; 2 Department of Vision and Imaging Technologies, Fraunhofer HHI, Berlin, Germany; 3 Visual Computing Group, Humboldt-Universität zu Berlin, Berlin, Germany; 4 Clinic of Cognitive Neurology, University Hospital Leipzig, Leipzig, Germany; 5 MindBrainBody Institute at the Berlin School of Mind and Brain, Humboldt-Universität zu Berlin, Berlin, Germany

**Keywords:** Neuroscience, Cognitive neuroscience, Visual system

## Abstract

Artificially created human faces play an increasingly important role in our digital world. However, the so-called uncanny valley effect may cause people to perceive highly, yet not perfectly human-like faces as eerie, bringing challenges to the interaction with virtual agents. At the same time, the neurocognitive underpinnings of the uncanny valley effect remain elusive. Here, we utilized an electroencephalography (EEG) dataset of steady-state visual evoked potentials (SSVEP) in which participants were presented with human face images of different stylization levels ranging from simplistic cartoons to actual photographs. Assessing neuronal responses both in frequency and time domain, we found a non-linear relationship between SSVEP amplitudes and stylization level, that is, the most stylized cartoon images and the real photographs evoked stronger responses than images with medium stylization. Moreover, realness of even highly similar stylization levels could be decoded from the EEG data with task-related component analysis (TRCA). Importantly, we also account for confounding factors, such as the size of the stimulus face’s eyes, which previously have not been adequately addressed. Together, this study provides a basis for future research and neuronal benchmarking of real-time detection of face realness regarding three aspects: SSVEP-based neural markers, efficient classification methods, and low-level stimulus confounders.

## Introduction

Virtual humans are becoming more and more common in a wide range of fields, such as film, video games, education, and virtual communication. However, a comprehensive theory of how the human brain perceives the difference between highly realistic virtual agents and real humans is still missing. Meanwhile, the human face may be the most informative interface during daily social interactions. Previous work suggested that the human brain decrypts aspects of facial information, such as emotional expression^1,2^, familiarity^3^, personal identity^4,5^, configurational information^6^ and, last but not least, the naturalness or "realness" of a face^7^. Recent advances in AI technologies of computer graphics (CG), especially some deep-learning-based methods such as generative adversarial networks (GANs) and variational autoencoders (VAEs), show the great potential of applying computer-generated images in different applications^8,9^. Specifically, the "Deepfake" technology has attracted extensive concerns for the ability to create hyper-realistic videos, and human observers can hardly realize its fakeness^10,11^. However, computer-generated virtual characters do not always receive positive feedback from the users.

One prominent phenomenon relating to the perception of artificially generated face images is the uncanny valley (UV) effect. The UV effect describes the phenomenon that highly human-like but not perfectly real virtual agents can be perceived as eerie and unappealing^12–14^. This phenomenon was firstly introduced by Mori in the 1970s, where he found a nonlinear relation between the degree of human-likeness of replicas and the dimension of *shinwakan* (this Japanese term is typically translated as *affinity* or *familiarity*)^12^. Although other studies interpreted this term also in alternative ways^15^, we here refer to the term *affinity*, as was also used by Mori in his latest article in 2012^12^. To be more specific, usually humans perceive the human replicas to be more comfortable when the human-likeness of humanoid objects increases, until some highly realistic but not perfectly human-like face images may lead to a “valley” on the dimension of affinity. Although the UV effect was commonly observed in a variety of tasks, diverse ways of generating stimuli, the variation across participants, and other variables might lead to different UV effect curves^16^. For instance, Złotowski et al. (2015) suggested that repeated interaction with robots may also affect the shape of the UV curve^17^. In recent years, many studies tried to reach a maximized level of perceived realness when they developed new technologies of CG taking into account the complex relationship between human likeness and affinity^14^. A number of theories aim to explain the UV effect, such as categorical uncertainty^18^, violation of prediction^19,20^, mind perception^21^, and pathogen avoidance^22^. However, most of these theories remain rather inconclusive as they cannot explain every uncanny object^23–25^. A recent meta-analysis, the first one on the UV effect to our knowledge, suggested that the specific type of stimuli and chosen affective indices for subjective ratings may play a decisive role for the UV effect^26^. Thus, studying the UV effect using objective brain measures, without relying on subjective ratings only, may advance the understanding of the UV effect.

Various studies assessed the perceived realness of virtual avatars via behavioral methods, such as subjective ratings scales^27,28^. Those subjective rating methods re-validated the widespread observation of the UV effect but could not always precisely predict whether one specific image falls into the “uncanny valley” or not, before requesting feedbacks from users. Moreover, the subjective rating is highly susceptible to various biases and can exhibit considerable variability over time. Recently, some researchers have started to use electroencephalography (EEG) as a neurophysiological tool to investigate the neural responses to real as compared to artificial images^29^. Typically, the N170, a negative component that peaks around 170 ms after stimulus onset at occipito-temporal recording sites, shows markedly larger amplitudes for face stimuli than non-face stimuli^30,31^. The modulation effect of the N170 was not only observed in the simple comparison of face stimuli as compared to non-face stimuli, but also in other face perception tasks. For instance, the amplitudes of the N170 show significant differences between inverted faces and upright faces^32^, as well as face images with different emotions^30^, facial movement in general^33^, and gaze directions^34–36^. A recent study suggested that the amplitude of the N170 component was modulated by face-realism^37^. In this study, the authors used face images of six levels of stylization as the stimulus set and they found a U-shaped modulation effect between the realness and the amplitudes of N170 components, with the largest neural responses for most abstract and most realistic images. Meanwhile, this study also found that the late positive potential (LPP) increased almost linearly with face realism. These results may thus represent first evidence for the UV effect from an ERP perspective. Another recent study successfully distinguished highly realistic AI-generated human faces and real human faces by decoding EEG^7^. These decoding differences were present even when users did not consciously report those differences. This study thus suggests that the EEG-based approach could serve as reliable feedback to improve the generation of computer graphics even when the users can barely tell the differences.

Apart from the event-related potential (ERP) technique, another widely used paradigm for studying the brain-electric correlates of face perception is based on steady-state visual evoked potentials (SSVEP). SSVEPs are neural responses evoked by periodic visual stimulation with a fixed frequency. These responses are typically generated in the visual cortex and adjacent regions^38,39^, exhibiting a relatively high signal-to-noise (SNR) ratio^40^. SSVEP contain responses at the stimulation frequency and its harmonics, which provides a convenient way to test the sensitivity of the visual system to different visual stimuli. SSVEP has been applied to determine image^41–43^, suggesting that SSVEP components can indeed be modulated by low-level yet complex details of the stimulus material. Moreover, similar inverted-face modulation effects as for the N170 component were found in SSVEP components^44–46^, located in the right visual cortex. Overall, those studies supported the plausibility of studying face perception processes with SSVEP. In the context of the UV effect, a recent study selected SSVEP as the neural marker of perceiving realness of computer-rendered faces^47^, using the same stimuli as the above-mentioned study on face-realness-related N170 effects^37^. Those face images were generated with six stylization degrees and three kinds of emotional face expressions^48^. As the first study to look at the modulation effect of stylized images with the SSVEP paradigm, that study found a negative correlation between subjective realness ratings and SSVEP amplitudes at the stimulation frequency of 5 Hz and its odd harmonics^47^.

However, limitations remained in this SSVEP-based study regarding the localization of the effects and the specificity of realness-related biomarkers. For instance, only one channel (Oz) was analyzed, which neglects the spatial information and the lateralization phenomenon of brain regions involved in face perceptions. Therefore, in order to (i) provide more neurophysiological insight, (ii) to explore the multivariate nature of SSVEP neuronal signals, (iii) to control for low-level features of visual stimuli, and (iv) to develop machine learning algorithms for a quick detection of realness levels, we reanalyzed the dataset presented in Bagdasarian et al. (2020)^47^.

## Methods and materials

### Participants

Ten subjects (two females and eight males, age range from 21 to 31 years) participated in the experiment. All participants had normal or corrected-to-normal vision and received financial compensation after the experiment. Informed consent from all subjects and/or their legal guardian(s) have been collected for study participation and publishing their results online by open-access publication. The experiment was approved the ethics committee of Technische Universität Berlin. All methods were performed in accordance with the guidelines and regulation at Technische Universität Berlin. The experimental procedures consisted of two parts: a behavioral part (subjective ratings of the stimuli) and a neurophysiological part (EEG assessment).

### Stimuli

**Figure 1:**
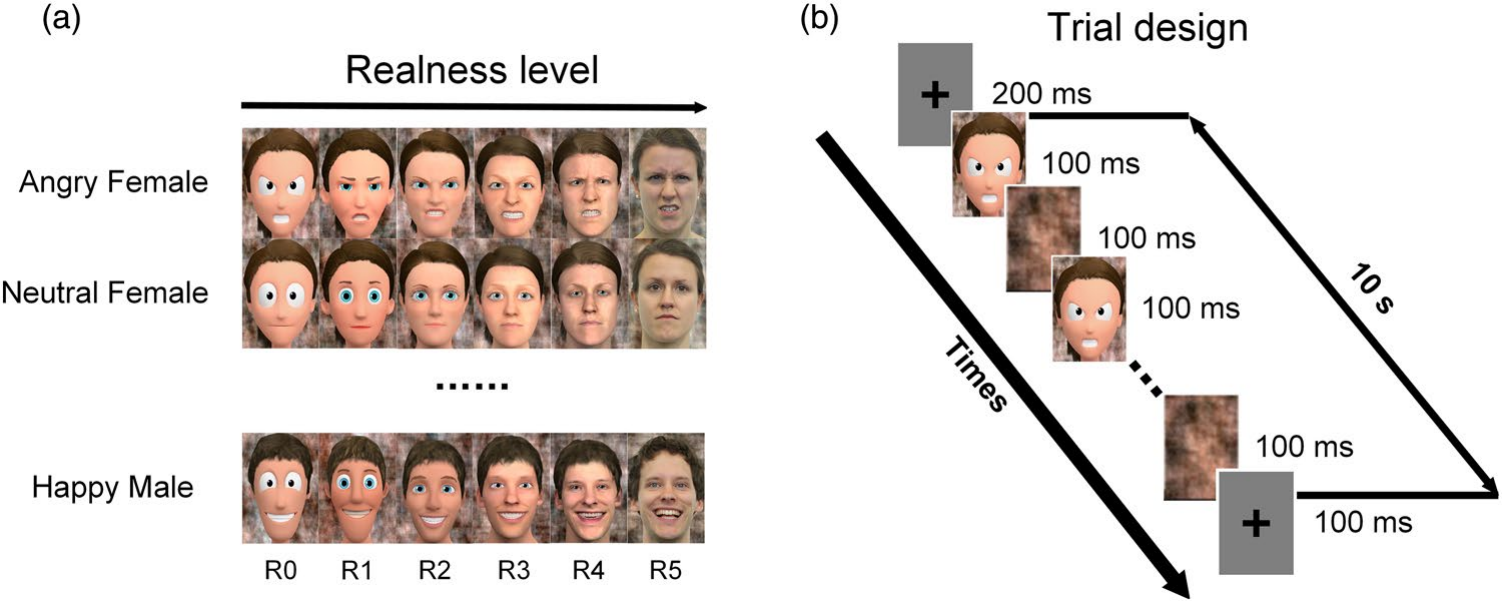
Stimulus set and experimental design. (**a**) The stimulus set, including 36 face images of different stylization levels and emotions, the backgrounds were the scrambled versions of the original images. From R0 to R5, the realness of images increased. (**b**) Trial design. Every session consisted of 36 trials, corresponding to 36 images in panel (**a**), the main stimulation frequency was set at 5 Hz. Every trial lasted 10 s every periodic stimulus process was repeated every 200 ms that contained 100 ms presence of face images and 100 ms of scrambled backgrounds.

A set of face images with different stylization levels was used as the stimulus material, based on a developed approach that is capable of creating a continuum with increasing degrees of realness^48^. Stimulus images had a size of 700 × 1000 pixels and were displayed on the center of an LCD screen (LG OLED65E6D-Z) with a resolution of 3840 × 2160 pixels at a viewing distance of participants of 1.2 m. In total, 36 face images (see Fig. 1a) were generated with six levels of stylization (R0-R5), two genders (male and female), and three emotions (neutral, happy, angry). From R0 to R5, as more details and complicated textures were integrated, the face images turned to be closer to authentic human faces. Moreover, to exclude potential side effects, backgrounds were replaced by phase-scrambled versions of the original images. Furthermore, we used Adobe Photoshop 2020 to manually measure the size of the eyes and the averaged luminosity of the face region of the stimuli, in order to control for these low-level visual features. The regions of eyes were automatically determined by the Adobe Photoshop.

### Experimental procedure

In the behavioral task, 36 face images were randomly presented to all participants, and the participants were asked to rate the shown images on five perceptual dimensions (appeal, reassurance, realism, familiarity, attractiveness) from 1 to 7^47^, among which realism was supposed to be the most relevant index for the current study. More details related to the definition of parameters and scales can be found in Bagdasarian et al. (2020)^47^. The subsequent EEG part consisted of eight sessions, each session lasted about seven minutes, and included 36 trials of ten seconds. In each trial one image of the stimulus set was selected in a random sequence, with each stimulus image repeated eight times across sessions. For two participants (S4 and S9) one session and for one participant (S5) two sessions had to be excluded due to loud noise next to the lab during the experiment. All trials started with a gray background screen presented for 200 ms. As the 4–8 Hz range was reported to be the optimal range for the SSVEP face discrimination task^49,50^, the primary stimulation frequency of face images was set to 5 Hz, so that face images were repeatedly shown every 200 ms with a duration of 100 ms, followed by scrambled background images with a duration of 100 ms, thus resulting in a 10 Hz reversing frequency between faces and backgrounds. After presenting the steady stimuli for ten seconds, all trials ended with a 100 ms gray screen (see Fig. 1b).

### EEG preprocessing

EEG data were recorded with a 64-channel Brain Products ActiCap, BrainAmp amplifier, and Brain Vision recording software with a sampling rate of 1 kHz. The electrodes were placed according to the standard 10–10 system. The ground electrode was AFz and the reference was FCz. Impedances were kept below 10 kΩ. More details can be found in Bagdasarian et al. (2020)^47^. Data were processed in MATLAB R2022b using the EEGLAB toolbox^51^. Topography figures were plotted with the MNE-python package^52^ in Python. Each trial lasted ten seconds, but the first second and the last second of raw data were cut to guarantee that the extracted EEG segments did not contain any ramp-up or ramp-down effects of the SSVEP signals. Thus, in the following parts of this article, these eight seconds of the data ([1 s, 9 s] of raw data) were used for analysis. Finally, a zero-phase third-order Butterworth filter with a passband from 3 to 40 Hz was applied (for the ERP analysis and TRCA classification).

The previous study with this dataset mostly emphasized findings at channel Oz^47^. In our study, to take advantage of the multi-channel data, we used spatio-spectral decomposition (SSD)^53^ as the preprocessing method of spatial dimension reduction. SSD is a spatial filter approach that aims to maximize the power of certain frequency bands while suppressing the power of flanking frequency bands^53^. SSD was performed on the concatenated raw EEG data of every single participant. As the major evoked oscillation in this study was at 5 Hz and its harmonics, components of [4 Hz, 6 Hz] in the raw data were considered as the signal part, while [2 Hz,3 Hz] and [7 Hz, 8 Hz] were defined as the noise part. After that, three primary spatial filters (i.e., sets of weights for each EEG channel) corresponding to the three largest eigenvalues (larger than 0.7) were selected. SSD patterns were reconstructed according to the approach presented in Haufe et al. (2014)^54^, on the basis of the covariance matrix of the narrow-band-filtered signals multiplied by the spatial filter. Because the SSD patterns have an arbitrary unit and different polarity for different participants, all SSD patterns were standardized so that the polarity was positive at channel Oz. Considering that different components may be generated by different sources and the three SSD components were only sorted based on the eigenvalues rather than their sources, we selected those SSD components whose patterns were maximally similar across participants. Based on the definition of error between original patterns and reconstructed patterns in Nikulin et al. (2011)^53^, the similarity was defined as the absolute value of the dot product of two normalized SSD patterns each.

As an alternative approach to use ERP-like signals in the time domain, the 200 ms EEG segments after each stimulus onset were considered as transient neural activities evoked by each visual stimulus. Among those transient responses, the N170 potential is the most obvious face-related ERP component. As N170 responses and SSVEP responses could be located in different areas according to the previous studies^37,47^, we chose Oz as the primary analysis channel for SSVEP responses and PO8 for the N170 potential. Furthermore, we also chose nine channels (Pz, PO3, PO7, PO4, PO8, POz, O1, Oz, O2) in the parieto-occipital region commonly used in SSVEP-based studies^55^ for an electrode cluster analysis. During the classification procedure, the data were down-sampled to 250 Hz to mitigate the potential issue of overfitting for each session in each trial. 200 ms EEG data repeated forty times were averaged for the ERP analysis. Additionally, we assessed the power spectral density over long EEG segments with the function *pwelch()*. FFT amplitudes of single trials were calculated with the function *fft()* with a Hamming window. The amplitude of the N170 component was defined as the mean value between 150 and 190 ms after stimulus onset. The scripts are available at https://github.com/Chen-YongHao/SSVEP-face.git.

### Classification

Task-related component analysis (TRCA), a classic spatial filtering method typically applied in SSVEP-based BCI^55,56^, was employed in this study to classify stylization levels of stimuli based on the EEG responses. With applying spatial filters on multi-channel EEG signals, TRCA aims to extract stimulus-event-locked signals through enhancing the SNR of repeated components. Following the previous studies on SSVEP^55,57^, we focused on the nine channels in the parieto-occipital region for the classification (Pz, PO3, PO7, PO4, PO8, POz, O1, Oz, O2).

The whole classification pipeline can be divided into two parts: model training and testing. At first, spatial filters were trained for each different class to maximize the inter-session covariances, thus maximizing the SNR of task-related (i.e., phase-locked) components. First, the averaged epoch was acquired as a template. The training data can be considered as $x\in {R}^{{{N}_{class}\times N}_{c}\times L\times {N}_{s}}$ and testing data ${x}_{test}\in {R}^{{N}_{C}\times L}$, where *N*_*class*_ is the number of classes, *N*_*C*_ is the number of selected channels, *L* is the number of sampling points, and *N*_*S*_ is the number of sessions (equal to 8 for most participants in this study). The optimized spatial filters for one class ${{w}_{TRCA}}^{({i}_{class})}, {i}_{class}\in \left[1,{N}_{class}\right]$ were acquired by:

1 $$\begin{array}{c}{w}_{TRCA}=\underset{w}{{\text{argmax}}}\frac{{w}^{T}Sw}{{w}^{T}Qw}\end{array}$$

where the matrix $S={({S}_{i,j})}_{1\le i,j\le {N}_{C}}$ was calculated through the sum of covariances across all possible combinations of sessions:

2 $$\begin{array}{c}{S}_{i,j}=\sum_{\begin{array}{c}{h}_{1},{h}_{2}=1\\ {h}_{1}\ne {h}_{2}\end{array}}^{{N}_{s}}{\text{Cov}}( {x}_{i}^{\left({i}_{class},{h}_{1}\right)},{x}_{j}^{\left({{i}_{class},h}_{2}\right)})\end{array}$$

**Figure 2:**
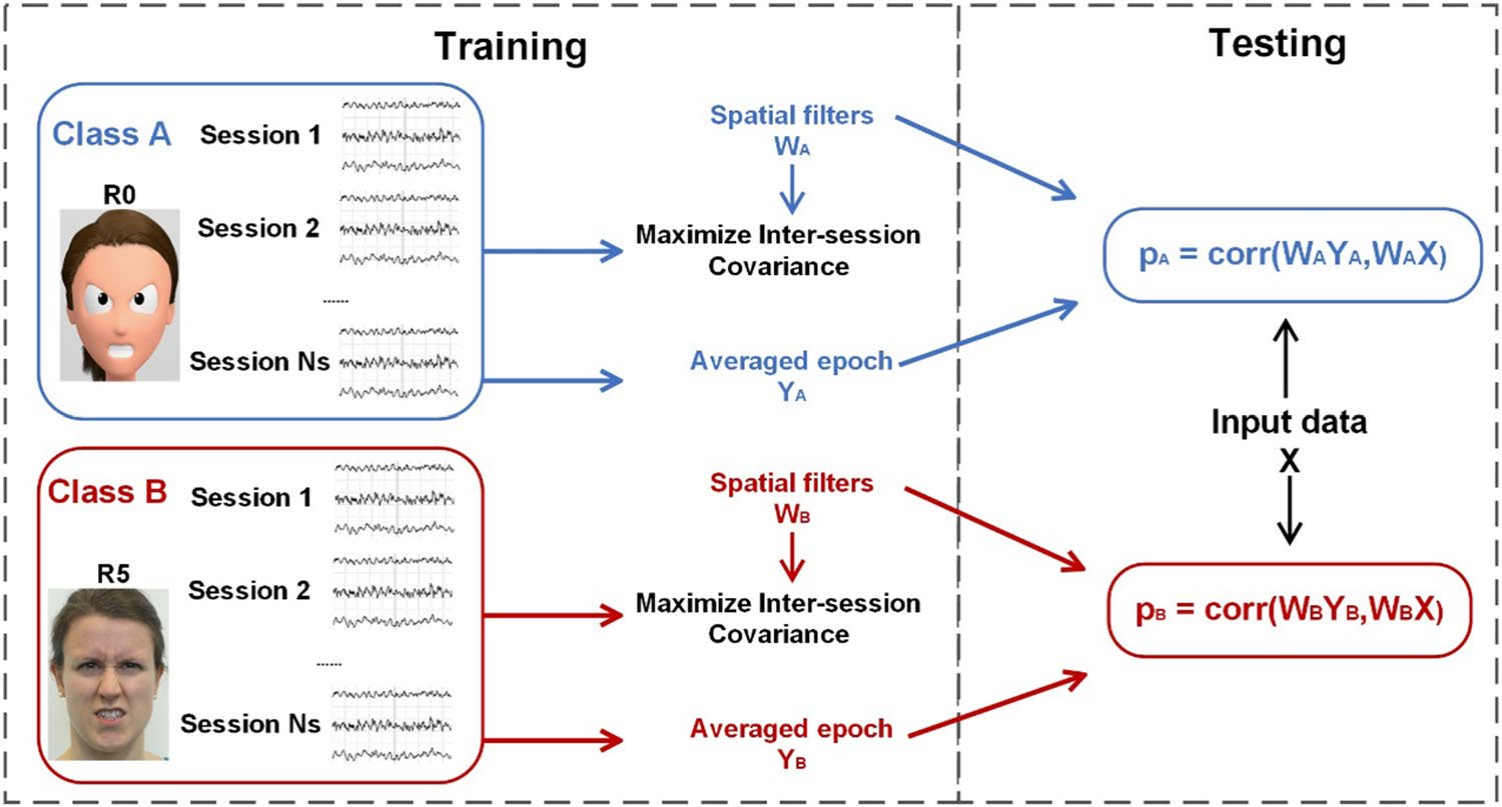
Framework of the TRCA-based classification algorithm (here shown for the two classes: R0 and R5). During the training process, independent spatial filters were trained to extract the task-related components by maximizing the inter-session covariance for each class. Those spatial filters were used to filter the multi-channel EEG signals and averaged training data across all sessions. Finally, the Pearson correlation coefficients between filtered input data and filtered templates served as classification metric, i.e., the class producing the largest correlation value was the classified result.

The matrix $Q={({Q}_{i,j})}_{1\le i,j\le {N}_{C}}$ was defined as:

3 $$\begin{array}{c}{Q}_{i,j}= Cov\left({{ \overline{x} }_{i}}^{({i}_{class})},{{\overline{x} }_{j}}^{({i}_{class})}\right)\end{array}$$

where the templates $\overline{x}\in {R }^{{{N}_{class}\times N}_{c}\times L}$ were the averaged training data of each class across all sessions. After *N*_*class*_ kinds of spatial filters ${{w}_{TRCA}}^{({i}_{class})} \in {R}^{{1\times N}_{c}}, {i}_{class}\in \left[1,{N}_{class}\right]$ were trained, the Pearson correlation coefficients between filtered templates and filtered testing data were calculated as the metric of classification. The data for testing were ${x}_{test}\in {R}^{{N}_{c}\times L}.$ The class corresponding to the largest correlation coefficients was chosen as the detection result: (Fig. 2)

4 $$\begin{array}{c}target = \underset{{i}_{\mathit{class}}}{{\text{argmax}}}\rho ({{w}_{TRCA}}^{\left({i}_{class}\right)}{\overline{x} }^{\left({i}_{class}\right)},{{w}_{TRCA}}^{\left({i}_{class}\right)}{x}_{test})\end{array}$$

To compare the discrimination between different classes, two kinds of pairs were selected. The first pair is R0 and R5, which has the strongest divergence in the appearance of stimuli images. We also used a second pair, R4 and R5, which has the lowest divergence in the appearance. Additionally, six classes from R0 to R5 were also selected as separate categories for global classification. Among those categories, the emotion states and the gender information remained balanced. The classification accuracies were averaged across all subjects, all emotion states, and both genders. We used the leave-one-out cross-validation method to build the training sets and the testing sets. Given that TRCA has been reported to perform well in SSVEP-BCI, even with very short data^55^, we chose both 2 s ([1 s, 3 s] of raw data) and 8 s data ([1 s, 9 s] of raw data) for the evaluation of accuracy under different conditions of data length. The data for training and testing always stayed balanced (angry female, angry male, etc.), when considering all included single trials.

### Statistical analysis

To statistically assess the non-linear relationship between realness levels and SSVEP as well as N170 amplitudes, we compared the model fit of linear regression models and quadratic regression models using the Akaike information criterion (AIC), Bayesian information criterion (BIC) as well as the model likelihood. The likelihood ratio test (LRT) served to statistically compare the likelihood of different models (using the χ^2^-test statistic). To account for the within-subjects experimental design, we used linear mixed-effect models (LMM) as the regression method with the lme4 package^58^ in R^59^. The lme4 model syntax was implemented as follows:

$$\begin{gathered} {\text{EEG response amplitude }}\sim { 1 } + {\text{ realness }} + \, \left( {{1 }|{\text{ subject}}} \right) \hfill \\ {\text{EEG}}\;{\text{response}}\;{\text{amplitude}} \, \sim \, 1 + {\text{realness}} + {\text{I}}({\text{realness}}^{2} ) + (1|{\text{subject}}) \hfill \\ \end{gathered}$$

Here EEG responses included SSVEP and N170, and the predictor was the level of realness (corresponding to the image categories from R0 to R5), which was quantified as 1 (R0) to 6 (R5). Participants were considered as random factor. As this experiment had a limited number of participants, we only considered a random intercept term ("1 | subject") across different participants in order to avoid over-fitting of random slopes. To quantify inter-subject variability, we used 95%-within-subject confidence intervals, as implemented in the *SummarySEwithin*() function in R, where the degree of realness was considered as the within-subject variable.

Paired t-tests were used to statistically compare the effects of confounding variables (eye size and luminosity in different image categories). For the correlation analyses, we used the *corr*() function and *partialcorr*() function in MATLAB. To further investigate the influence of our original predictors beyond the effects of confounding variables (e.g., eye size), we included the confounders in the mixed-effects models as follows:

$$\begin{gathered} {\text{EEG response amplitude }}\sim { 1 } + {\text{ realness }} + {\text{ confound }} + \, \left( {{1 }|{\text{ subject}}} \right) \hfill \\ {\text{EEG}}\;{\text{response}}\;{\text{amplitude }}\sim \, 1 + {\text{realness}} + {\text{confound}} + {\text{I}}({\text{realness}}^{2} ) + (1|{\text{subject}}) \hfill \\ \end{gathered}$$

In the classification part, permutation tests were employed to assess classification performance. For two-class classification, the trials of two classes were randomly permuted and fed into the training algorithm. After being repeated for 1000 times, the *p*-value was calculated as the proportion of sampled permutations where the accuracy was greater than the real classification accuracy. The idea was similarly applied in the six-class classification procedure. For all analyses, the statistical significance level was set to *p* < 0.05.

## Results

### SSVEP amplitudes

**Figure 3:**
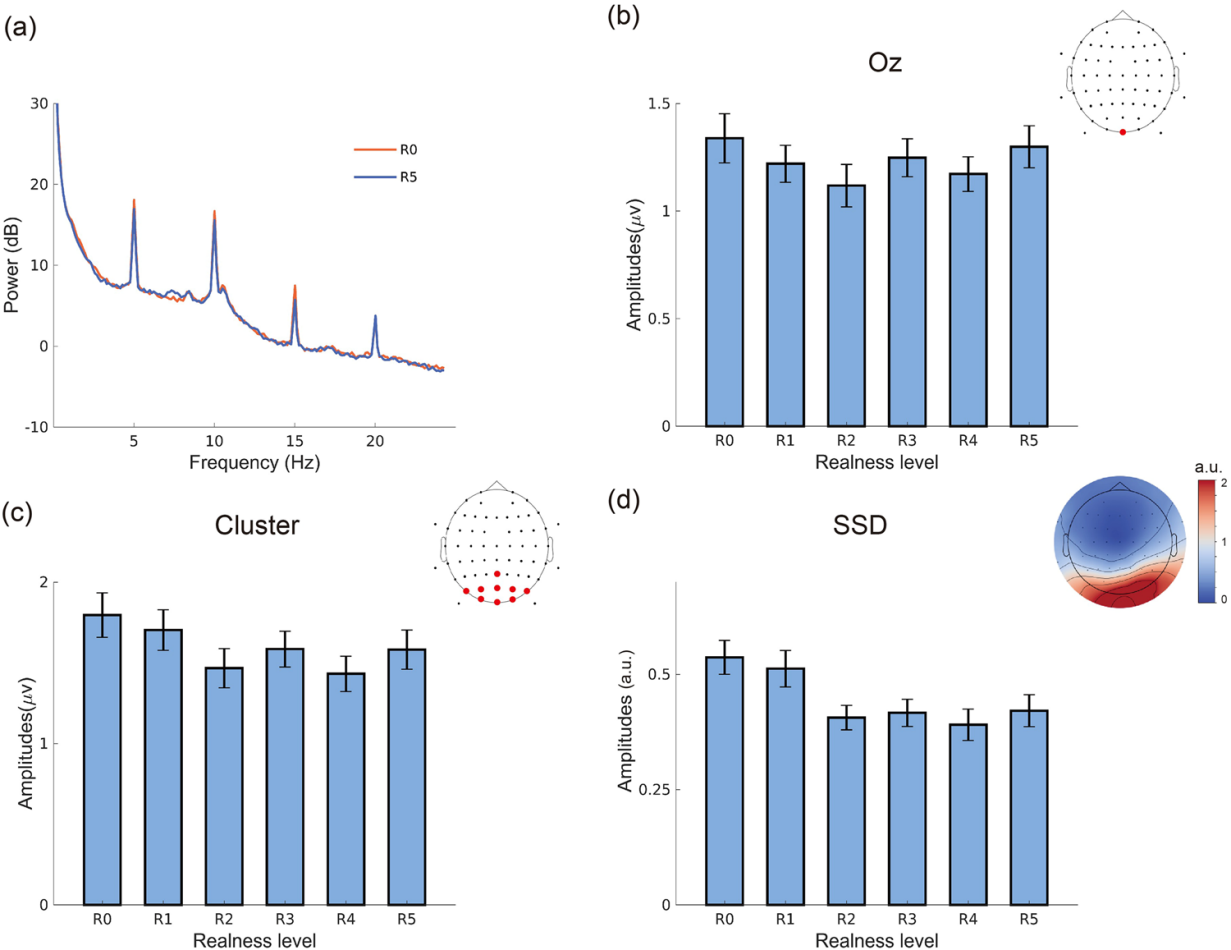
SSVEP results. (**a**) Log-transformed spectral power of averaged responses across all participants and all trials (at electrode Oz). (**b**) FFT amplitudes of 5 Hz across different realness levels (at electrode Oz). (**c**) FFT amplitudes of 5 Hz across different realness levels (cluster of parieto-occipital region electrodes). (**d**) FFT amplitudes of 5 Hz across different realness levels (after applying the SSD approach), the y-axis is arbitrary unit. Amplitudes in (**b**)(**c**)(**d**) were averaged across all participants and all sessions. The error bars show the 95% within-subject confidence intervals. The topographies in (**d**) show the averaged SSD pattern, the unit is arbitrary unit.

Neural responses to face stimuli of different stylization levels were assessed using SSVEP amplitudes. According to the power spectrum presented in Fig. 3a, SSVEP responses peaked at 5 Hz (stimulation frequency) and its harmonics. Similar to a previous study on visual ERP^60^, we found a nonlinear relationship between the degree of realness and SSVEP amplitudes at 5 Hz. Although not particularly pronounced at SSVEP level (in contrast to N170, results presented below), this finding appeared consistent when extracting SSVEP amplitudes from one channel (Oz), from a parieto-occipital electrode cluster, and also when performing a spatial filtering approach using SSD, tailored to detect periodic signals at 5 Hz (Fig. 3). This was confirmed statistically with LMM comparisons, where the quadratic regression model always showed a significantly better model fit than the linear model according to LRT (χ^2^ = 8.859, *p* = 0.003 for Oz; χ^2^ = 10.737,* p* = 0.001, for the parieto-occipital electrode cluster; χ^2^ = 16.733, *p* < 0.001 for the SSD approach). Furthermore, the AIC and BIC of the quadratic models were always significantly lower than for the linear models (Table 1), indicating better model fits. Overall, these results suggest that the most realistic face images and the most abstract face images evoke higher SSVEP responses than medium levels of realness, matching the "valley" phenomenon of the UV hypothesis. However, when considering harmonics at 10 Hz and 15 Hz, we could not find similar effects in the comparison of linear models and quadratic models (*p* > 0.05 for amplitudes at 10 Hz and 15 Hz, both at channel Oz and in the parieto-occipital electrode cluster).

**Table 1:** The relation between EEG response amplitudes and the degree of realness.

Signal	Channel	Model (L/Q)	AIC	BIC	Log-likelihood	LRT
SSVEP	Oz	L	485.76	501.30	− 238.88	
		Q	478.90	498.33	− 234.45	χ^2^ = 8.859,* p* = 0.003*
	Cluster	L	310.94	326.48	− 151.47	
		Q	302.20	321.63	− 146.10	χ^2^ = 10.737, *p* = 0.001*
	SSD	L	876.46	892.00	− 434.23	
		Q	861.72	881.15	− 425.86	χ^2^ = 16.733, *p* < 0.001*
N170	PO8	L	957.08	972.63	− 474.54	
		Q	942.78	962.21	− 466.39	χ^2^ = 16.305,* p* < 0.001*
	Cluster	L	933.97	949.41	− 462.98	
		Q	915.41	934.84	− 452.70	χ^2^ = 20.558, *p* < 0.001*

L and Q refer to the linear and quadratic models, respectively. LRT is the likelihood ratio test. Akaike information criterion (AIC), Bayesian information criterion (BIC) are indices of model fit. Likelihood is the log transformed likelihood. The regression models with lower AIC, lower BIC, and higher likelihood indicate a better model fit. SSD referred to the clustered SSD results after applying optimal SSD filters (*Significance level: *p* < 0.05).

### ERP measurement

**Figure 4:**
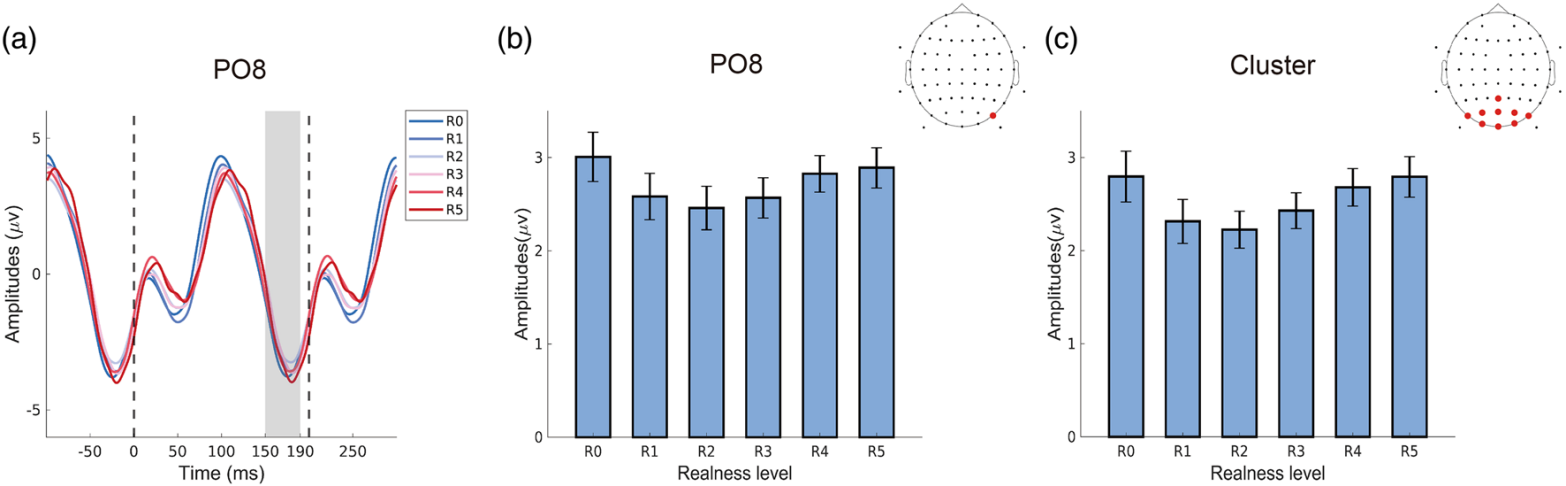
ERP-like components. (**a**) Grand average of the ERP-like response (at electrode PO8) The black dotted lines indicate the stimulus onsets and the grey area marks the time window for calculating the amplitudes of N170-like components. (**b**) Extracted response magnitudes of the N170-like ERP component at electrode PO8. (**c**) Extracted response magnitudes of the N170-like ERP component in the parieto-occipital electrode cluster. The larger bars indicate larger responses (negative N170 amplitudes were inverted). The error bars show the 95% within-subject confidence intervals. The topographies show the location of channel PO8 and the parietal-occipital electrode cluster.

Usually, it is not feasible to extract ERP components in high-frequency SSVEP paradigms because stimuli are presented with short inter-stimulus intervals. However, taking advantage of the rather slow stimulation frequency of 5 Hz in the current paradigm, the averaged 200 ms responses after each stimulus onset could be investigated not only in the frequency domain but also in the time domain. Indeed, N170-like components were observable in the ERP, as shown in Fig. 4a. First, as the N170 is one of the most important ERP components in face perception, we measured its amplitudes by calculating the mean value between 150 and 190 ms after stimulus onset. Because N170 components were in the negative range, the inverse values were used to indicate response magnitudes in Fig. 4b and c. Correspondingly, larger bars indicate larger responses. Similar to the results in the SSVEP analysis, amplitudes of the N170 also exhibited a quadratic relationship, well in line with the result of a previous study^60^. Both at electrode PO8 and in the parieto-occipital electrode cluster, the modulation by realness levels were better characterized by the quadratic than linear effect terms (χ^2^ = 16.305, *p* < 0.001 for PO8; χ^2^ = 20.558, *p* < 0.001 for parieto-occipital electrodes cluster). Moreover, the quadratic models showed lower AIC and BIC values compared with the linear models, further supporting their better model fit (Table 1). Moreover, the latency between the N170 peak and the stimulus onset varied slightly (channel PO8, R0: 173.1 ± 2.6 ms, R1: 176.9 ± 1.9 ms, R2: 175.5 ± 2.6 ms, R3: 178.9 ± 2.4 ms, R4: 177.51 ± 2.7 ms, R5: 177.38 ± 2.7 ms). The two-way ANOVA post hoc test results indicated that the only pair with a significant difference in latency is R0 & R3 (*p* = 0.023; Bonferroni-corrected).

### Spatial distribution

**Figure 5:**
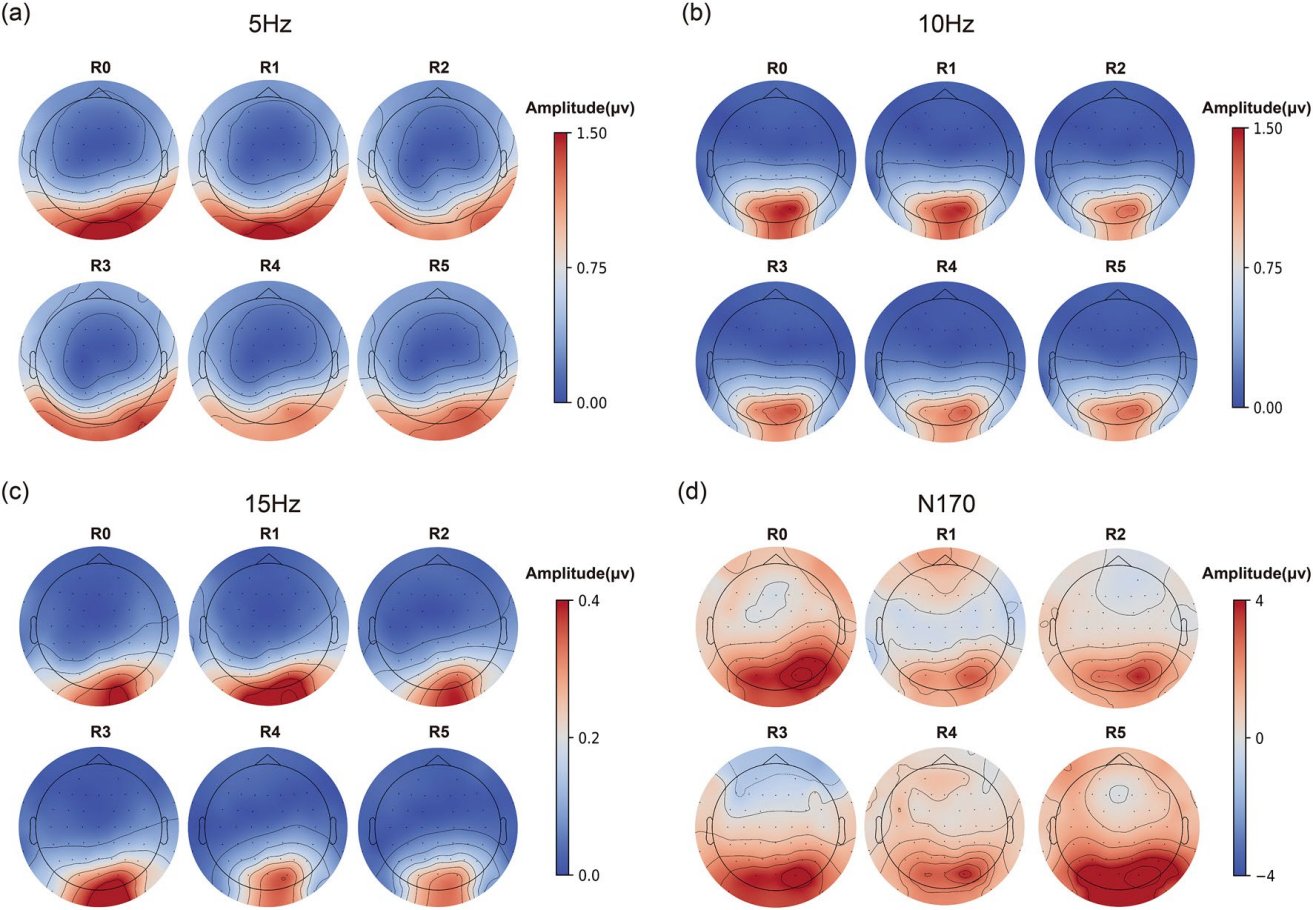
Scalp topographies of SSVEP and N170 responses across the six realness levels. (**a**) Amplitudes of 5 Hz SSVEP components. (**b**) Amplitudes of 10 Hz SSVEP components. (**c**) Amplitudes of 15 Hz SSVEP components. (**d**) Amplitudes of N170-like components. Please note the polarity inversion for N170 amplitudes in panel d. In all panels, amplitudes were averaged across all participants and sessions.

Figure 5 shows the scalp topographies of FFT amplitudes at 5 Hz and its harmonics (10 Hz, 15 Hz), and amplitudes of N170-like components. We found that SSVEP responses, both at the fundamental frequency and its harmonics, were located in the occipital lobe with a visible lateralization towards the right hemisphere. The N170-like components had a parieto-occipital topography as well, with a corresponding right-hemisphere lateralization in agreement with previous EEG studies on face processing^32,61,62^. Most of these activities extended laterally except for the 10 Hz responses, which had a more spatially focused pattern, possibly indicating rather early visual processing. Generally, the topographical distribution maintained relatively stable across different levels of realness, and only the response amplitudes varied across these stimulus categories.

### Confounding variables

**Figure 6:**
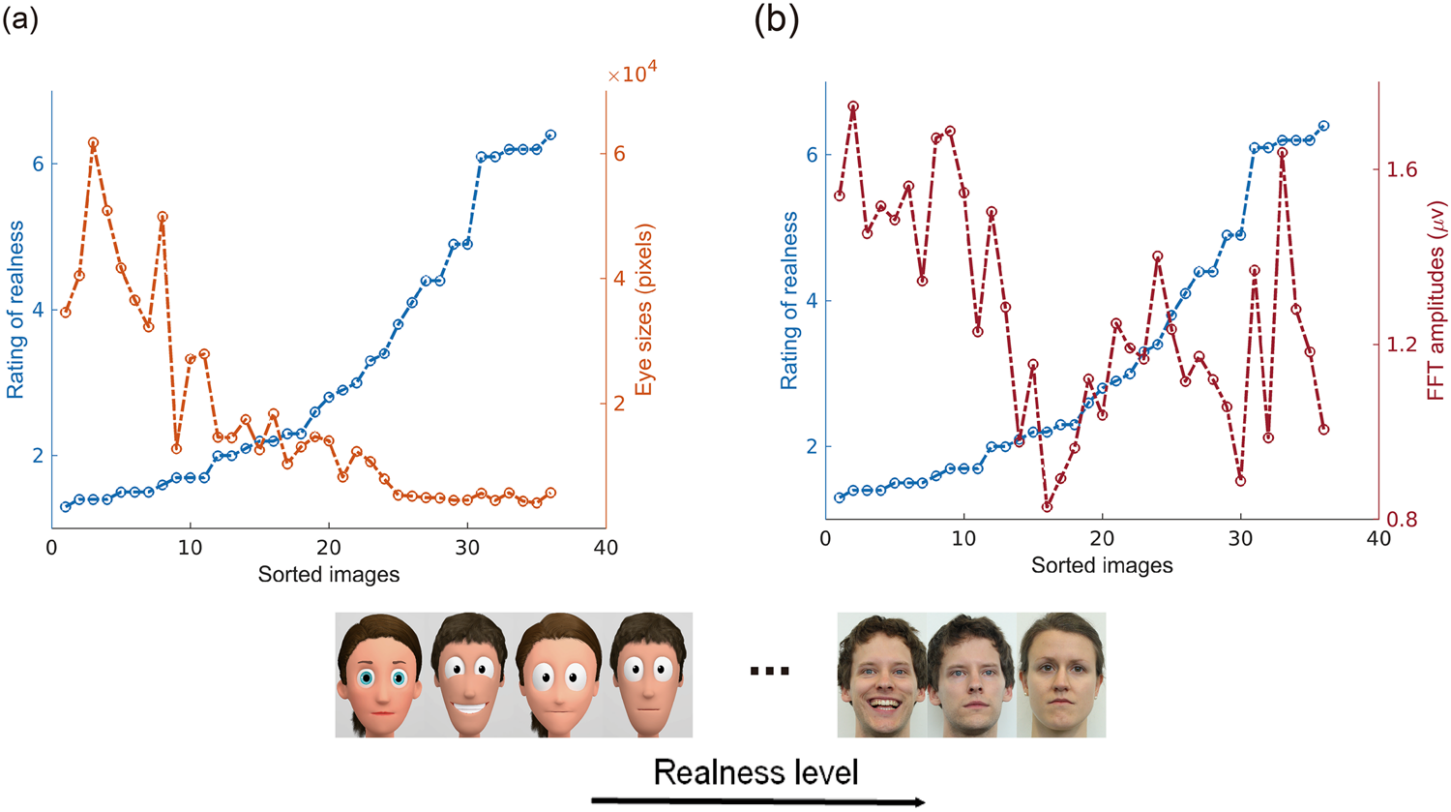
Interrelation of neural responses, realness ratings and confounding factor eye size. (**a**) Eye sizes (pixels) and the rating of realness. (**b**) FFT amplitudes of 5 Hz components (at electrode Oz) and the rating of realness.

The size of the eyes was one obvious low-level visual feature that varied across stylization levels, which may arguably affect the neural responses. As shown in Fig. 6, the mean value of eye sizes and the behavioral realness ratings were highly negatively associated (*r* = − 0.719, *p* < 0.001). However, taking the result of channel Oz for instance, the 5 Hz amplitudes were also highly negatively correlated with the rating of realness (*r* = − 0.353, *p* < 0.05). Thus, the question emerged whether our neural effects of realness levels were driven by such low-level visual features. We tested this, again comparing quadratic and linear mixed effects models, but now including the covariate term *eye size*. As shown in Table 2, quadratic models still showed the better model fit than linear models in all our comparisons. Importantly, it should be noted here that images of realness categories R4 and R5 did not significantly differ regarding their eye sizes (*t*(5) = − 0.309, *p* = 0.769), which gave us the opportunity to classify realness levels independently from eye size in the classification analyses presented below. As luminosity may also contribute to the modulation of SSVEP^63^, we assessed whether luminosity systematically differed across stylization categories. And this was not the case (*p* > 0.05).

**Table 2:** The relation between EEG response amplitudes and the degree of realness when controlling for the confounding factor eye size.

Signal	Channel	Model (L/Q)	AIC	BIC	Log-likelihood	LRT
SSVEP	Oz	L	486.45	505.88	− 238.22	χ^2^ = 9.535,
		Q	478.91	502.23	− 233.46	*p* = 0.002*
	Cluster	L	311.60	331.03	− 150.80	χ^2^ = 12.303,
		Q	301.30	324.62	− 144.65	*p* < 0.001*
	SSD	L	873.96	893.39	− 431.98	χ^2^ = 13.521,
		Q	862.44	885.75	− 425.22	*p* < 0.001*
N170	PO8	L	451.62	471.05	− 220.81	χ^2^ = 18.529,
		Q	435.09	458.40	− 211.54	*p* < 0.001*
	Cluster	L	537.15	556.58	− 263.58	χ^2^ = 20.634,
		Q	518.52	541.84	− 253.26	*p* < 0.001*

L and Q refer to the linear and quadratic models, respectively. LRT is the likelihood ratio test. Akaike information criterion (AIC), Bayesian information criterion (BIC) are indices of model fit. Likelihood is the log transformed likelihood. The regression models with lower AIC, lower BIC, and higher likelihood indicate a better model fit. SSD refers to the results after applying SSD filters (*Significance level: *p* < 0.05).

### Classification

**Figure 7:**
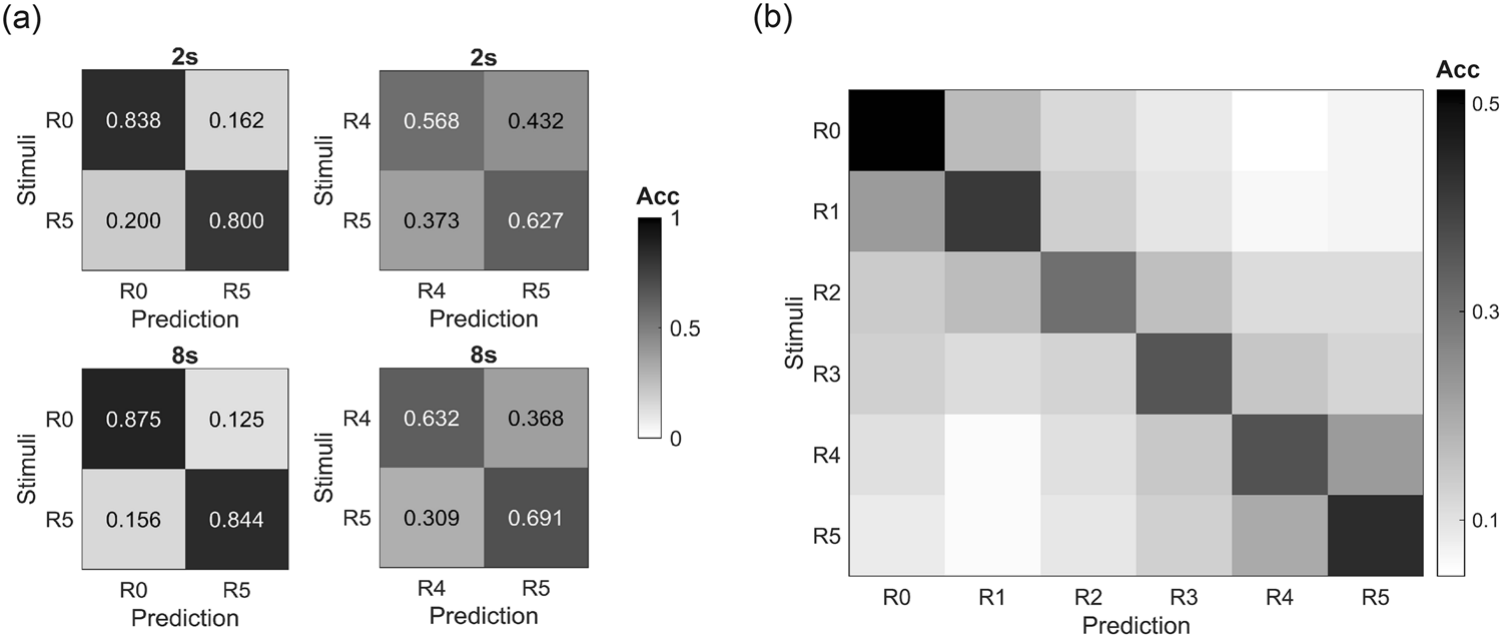
Normalized confusion matrix of the classification results. (**a**) Two classes (R0 and R5, R4 and R5). Numbers indicate the classification accuracy. (**b**) Six classes (R0 to R5). The average accuracy was 39.48 ± 9.58% for 2 s. A pronounced diagonal line (correct prediction for a given class) represents an effective classification. Acc indicates the averaged ratio of different classification outcomes.

We applied TRCA to classify realness levels of the face images using data of different length. As shown in Fig. 7, for the six-class (R0 to R5) classification task, the averaged accuracy was 47.46 ± 11.79% when all 8 s data were utilized. If the data was restricted to 2 s (window: 1 s to 3 s after stimulus onset), the average classification accuracy across all subjects and all emotion states was 39.48 ± 9.58%, which was still significantly higher than the chance-level (16.7%) of a six-class classification problem (*p* < 0.001). The confusion matrix, see Fig. 7b, demonstrated a pronounced diagonal line (correct prediction for a given class) where predictions were correct, which represents an effective classification. It can also be inferred that false detection most often occurred in the group of R4 and R5, likely because of high similarity between these two stimulus categories.

Additionally, we compared the two-class classification of stimuli with the smallest (R4 and R5) and largest difference (R0 and R5). We found that even with the highly similar pair R4 and R5, the average accuracies for 2 s (59.29 ± 7.19%,* p* = 0.014) and 8 s (65.80 ± 5.33%,* p* < 0.001) were still higher than chance-level. While for R0 and R5, the average classification accuracies were higher both for 2 s (81.70 ± 7.04%, *p* < 0.001) and 8 s (86.19 ± 7.39%, *p* < 0.001).

## Discussion

Using electroencephalography (EEG) in a paradigm of rapid presentation of face stimuli, the current study examined the neurophysiological underpinnings of realness perception in gradually stylized human face images. A previous study on this dataset mainly focused on the correlation between the amplitudes of SSVEP responses at one specific channel and behavioral data^47^. To further extend these findings, our study aimed to comprehensively explore neuronal processes reflecting realness perception. To this end, we analyzed neuronal responses in both the frequency and time domain by SSVEP and evoked responses (N170), respectively. We found that the amplitudes of neural responses, reflected both in SSVEP and N170 potentials, exhibited a quadratic relationship with the degree of realness. Although another previous study using the same stimuli material but a different paradigm has reported a similar quadratic relationship between N170 amplitudes and the level of realness^37^, there is no conclusive explanation why this phenomenon exists as it may also reflect low level visual features relating to different realness levels.

It is challenging to acquire pronounced and clean ERP if we only consider the transient response after the first stimulus onset due to the limited number of trials in this study. Therefore, we estimated the ERP-like responses by averaging all the 200 ms data segments to maximize the utilization of our data. In other words, in each trial, which consists of 8 s of data, we obtain the ERP by averaging 40 segments (8 s/200 ms). Taking into account the periodic presentation of the stimulus in the SSVEP paradigm, each 200 ms data segment might be affected by the previous stimulus, which is why we use the terms “N170-like” and “ERP-like” responses. Nevertheless, the baseline of SSVEP was obtained by a band-pass filter^64^ from 3 to 40 Hz. Importantly, the magnitudes around the stimulus onset do not differ significantly across stylization levels. As a result, the amplitudes of the estimated ERP responses, particularly the N170, should reflect the variations in activation levels of the human brain when perceiving these faces.

The N170 is an ERP component that has been repeatedly shown to reflect face processing^30^. Furthermore, many studies have found the N170 to be modulated by structural properties of face stimuli, such as emotional expression^31^, facial movement in general^33^, and eye gaze directions in particular^34–36^. In this context, it has been suggested that the N170 is generated by brain processes involved in the structural encoding of face stimuli^65^. Thus, such configurational analyses of the face’s features may be a critical driver for N170 amplitude effects. Another classical experiment in face perception showed that N170 amplitudes also increased when participants are presented with inverted faces^32,66^. In other words, the brain may need more "effort" to deal with the inverted situations, resulting in higher N170 amplitudes. Combining those two major points in the context of our study, we propose that, following the idea of N170 amplitudes being increased when participants were presented with inverted faces, the human brain may need more "effort" to recognize the images as genuinely human faces when being presented with the cartoon-like images, leading to highest amplitudes in the cases of most stylized images (R0 in this study). However, contrary to the typical findings from face-inversion experiments^32,66,67^, where the N170 latency is usually larger for stimulus categories requiring more “effort” to recognize them as a face, we did not observe a similar N170 latency shifting phenomenon for these highly stylized images. In fact, the N170 latency for R0 was the smallest (at least descriptively). This might be attributed to salient low-level features, such as the large eyes in R0, which may lead to a faster recognition of the stimuli as face-like structures^67^. At the same time, following the structural encoding hypthothesis^5^, more neural activity is evoked by an increasing number of facial details as the level of stimulus realness increases, such as by richer information on emotional face expressions or identity cues, which should in turn result in higher N170 amplitudes in the cases of real photos (R5). For the images with middle ranges of realness, the human brain may need to compromise between those two factors, which leads to the quadratic modulation effect. In addition to the above-mentioned explanations, the N170 amplitude has been previously linked to mechanisms of predictive processing, specifically to prediction errors, in several recent studies^66,68,69^. Thus, the impact of prediction violations regarding real-face features may also contribute to the higher N170 amplitude observed for the rather unnaturalistic stimulus categories. Besides, the non-linear relation between the realness and brain responses could be partially explained by the fluctuation of emotional arousal in the UV effects. That is, following the definition of the UV effect, images with different realness levels trigger inconsistent emotional feelings, meanwhile, the emotional arousal affects the general level of related responses (N170, e.g.). Actually, the emotional component of the UV effect was indeed observed in the behavioral results of Bagdasarian et al. (2020)^47^, where most participants reported that images of class R4 were more likely to evoke negative feelings (reflected as appeal; reassurance; attractiveness), compared with class R3 and class R5.

Although the SSVEP is conceptualized as a response to periodic stimuli, and the ERP as a response to individual stimuli, our study found compatible quadratic relationships between the amplitudes and the realness level, including both SSVEP and N170. Researchers often would not analyze the ERP responses in an SSVEP paradigm because of the high stimulus-presentation frequency that leads to the overlap of the current and preceding evoked response. However, benefiting from the 200-ms inter-stimulus interval in the current study, we here have the opportunity to fill the gap between previous studies that either found an effect of face realness on N170 amplitudes or SSVEP amplitudes. Our data suggest that these effects may originate from the same neuronal mechanism, that is hypothetically, the structural encoding of facial features. Moreover, SSVEP could be modeled as the temporal superposition of transient ERP^70^, which probably explains why we found similar quadratic modulation effects in SSVEP and N170 amplitudes. However, SSVEP responses are not only the superposition of N170 components but also of other ERP components, such as the P100. Presumably, the complexity of superimposed ERP responses in the SSVEP measure thus leads to the differences between the SSVEP and the isolated N170 component, as reflected in the more pronounced quadratic relationship for the N170 (Fig. 4) as compared to SSVEP (Fig. 3). Moreover, to further utilize the spatial information of the EEG and extend our analyses from the sensor-level, we applied SSD to extract the most pronounced and consistent SSVEP responses across subjects. Although also the SSD results demonstrated realness effects of a non-linear nature, we did not find a clearer quadratic relationship as compared to N170 results. Moreover, the nonlinear relationship after applying SSD was not identically equal to the sensor-level results. To be more specific, in contrast to the findings for a single channel (Oz), SSVEP amplitudes evoked by cartoonish faces (R0 & R1) were higher than those for real faces (R5) after implementing SSD. Given that the SSD results were based on a weighted summation of all sensors, this discrepancy may suggest that not necessarily the strongest but rather spatially specific neuronal activation is relevant for processing realistic as compared to cartoonish faces. Or, in other words, increased amplitudes for real faces observed at electrode Oz may thus reflect different neural sources compared to the activity that is extracted with the spatially separated SSD components. Despite the descriptive effect difference, our statistical analyses indicated a quadratic rather than a linear model fit for the *both* the sensor-level and SSD-derived neural signals. This further supports the idea that SSVEP includes a mixture of N170 and other visual evoked response components that might not all exhibit the same effect across realness levels. Nevertheless, given that we observed in-principle corresponding realness effects in both SSVEP and N170, an advantage of SSVEP is its rapid stimulation frequency, thus offering a less time-consuming but still informative way of probing neural correlates of a face stimulus´ realness.

Furthermore, compared with the components of the fundamental frequency (5 Hz), the harmonics might contain other additional information at higher frequency. We did not find a similar quadratic relationship for the amplitudes of harmonic components (10 Hz and 15 Hz). The even harmonics (i.e., 10 Hz, 20 Hz, etc.) might have been contaminated by the 10 Hz refreshing frequency between the stimuli and the backgrounds. In general, higher harmonics do not necessarily indicate the presence of evoked responses at higher frequencies. They may in fact rather reflect a non-sinusoidal nature of neuronal signals at the base frequency^71,72^. Importantly, higher harmonics are the first to be affected by the low SNR of the neuronal responses^72^ and thus are expected to demonstrate less significant or even absent statistical effects compared to the base frequency (i.e., in our case 5 Hz). Furthermore, according to the scalp topographies in Fig. 5, the 5 Hz component was extended towards lateral parieto-occipital regions as compared to the higher harmonics of the SSVEP. This suggests that the 5 Hz component spanned neural processes from early visual perception in medial occipital areas up to specialized face-related neural activity.

Besides, many low-level visual features, especially the eyes, may influence the amplitudes of both SSVEP and the N170. Although measuring the size of the white sclera to evaluate the influence of eye size is a well-established approach used in studies on real faces and correlates with brain activity^73^, we measured the size of the whole eye given that the sclera could not always be clearly extracted in our stimulus material (due to a cyan blue ring between the white sclera and the iris in some of the most cartoonish images). Nevertheless, our conclusion remains unchanged even when replacing the predictor eye size with the size of white sclera: we still find that the behavioral assessment of realness is negatively associated with the size of sclera (*r* = − 0.589, *p* < 0.001). There were no significant differences in either the eye size or sclera size between the images of R4 and R5 (*p* > 0.05). We found that eye size had a negative correlation with the degree of realness in our stimulus set. However, we showed that quadratic models describe EEG data better than linear models even after linearly regressing the eye size. Thus, the observed nonlinear relationships between EEG response amplitudes and realness levels were not driven by low-level stimulus features such as eye size. However, our findings strongly emphasize that such factors need to be carefully controlled in future studies, either already during stimulus preparation or by including eye size as a covariate in statistical models. Additionally, for the typical pair R4 and R5, in which we did not find any difference in eye size and luminosity, the classification algorithm successfully distinguished those two categories. We also found a significant difference in the N170 and SSVEP amplitudes, between the group of R4 and R5 (N170: *p* < 0.001, SSVEP: *p* < 0.001). Those results suggest that a comparison between highly realistic CG images and photos of real people is indeed possible with EEG to further explore how the human brain perceives face realness.

In our study, we implemented two kinds of spatial filtering methods: SSD and TRCA. SSD was chosen to focus on signals in the narrow frequency band around 5 Hz. In contrast, TRCA was chosen to focus on broad-band signals, phase-locked to the rapid stimulus presentation. TRCA was achieved by maximizing the cross-session covariances, leading to optimized spatial filters for "task-related" (i.e., stimulus-locked) activity. Another crucial factor affecting the overall classification performance is the length of the time window. In our study, we selected 2 s and 8 s to compare the classification accuracy in time windows of different lengths. For the pair of stimulus categories R4 and R5, the accuracy of 2 s data was smaller than 8 s data, while for the pair of R0 and R5, the accuracy did not show a significant difference between 2 and 8 s. In other words, the classification pair of R0 and R5 may need even fewer data to be classified. However, the classification between groups of R0 and R5 could be affected by other confounds, such as the large eye in the stimulus category R0, given that is known that the neural processing of face images is affected by this parameter^34–36^. Thus, contrasting the R4 and R5 categories may be most informative in the context of realness-related neural activity due to their comparability of low-level visual features (e.g., eye size)., Overall, we suggest that SSVEP-based classification may represent a paradigm that allows for saving experimental time (since it requires shorter data segments) compared to traditional ERP, in order to decode perceived realness levels from neural data.

It should be noted that the key idea of the classification algorithm was fully based on the Pearson correlation between filtered templates and filtered testing data. Thus, the classification results are based on a number of diverse spatial and temporal features of neuronal responses. This includes the effects of different amplitudes, the differences in scalp topography, and potentially also the variability of latencies of the SSVEP responses across stimulus conditions. These rich neuronal parameters allowed us to classify with EEG the realness level of face images. This may represent a promising starting point for future studies, further pinning down the neural substrates of realness perception, as it is still an open question how aforementioned complex EEG features interrelate with each other (e.g., spatial aspects, amplitude, phase, and other parameters). Another important factor in the classification approach is the way the channels are selected. In this study, because SSVEP components are usually located in visual cortical areas^40^ and to avoid overfitting, we chose nine channels in the parieto-occipital region as the first step of channel selection. However, a broader a-priori channel selection may be conceivable in future studies, too, for example to also be able to assess higher cognitive processes that happen further downstream of the neuronal response cascade. In general, this classification algorithm works well in the single-trial detection process. Thus, a potential application of this algorithm could be a real-time system that can quantify the realness level of face images by decoding the EEG data. A novel detection system that can detect realness levels according to immediate neural responses automatically might be helpful for the CG designer to better cross the "valley" in the UV phenomenon.

In conclusion, our study investigated how face images with different levels of stylization modulated the amplitudes of neural responses, including SSVEPs and the N170 component. We found a quadratic relationship between response amplitudes and the degree of realness, which may well correspond to the UV. Of note, face perception is a complex process, which certainly also entails additional neural activities. Taking the UV effect as an example, as suggested in a recent review^25^, ERP correlates of the UV effect may vary from early negative potentials (N170) to late positive potentials. Furthermore, the current study examined realness perception in a very wide range of realness levels (simple cartoon images to real photographs). To pinpoint the neural correlates of realness perception even further, it would be desirable to "zoom" into realness levels around the uncanny valley in future studies. For instance, would SSVEPs and N170 amplitudes show a similar relationship with the stimulus’ realness levels also with more subtle differences here and would this correspond to subjective realness perception? Moreover, it may be another promising research avenue to utilize such realness-perception correlates in the EEG to inform algorithms for realistic face image generation in a biologically meaningful way.

## Data Availability

The datasets acquired during the study are available to researchers upon reasonable request to the corresponding author and with appropriate institutional review board approval.
